# Supplementary material for: Architecture of the chromatin remodeler RSC and insights into its nucleosome engagement
Source: eLife. 2019 Dec 30;8:e54449. doi: 10.7554/eLife.54449 (PMC6959994; doi:10.7554/eLife.54449)
Supplement: Figure 2—source data 1. — Table of subunit homology for complexes in yeast and humans. [file elife-54449-fig2-data1.docx]

| **Species** | **YEAST** | | **HUMAN** | | |
| --- | --- | --- | --- | --- | --- |
| **Complex** | **RSC** | **SWISNF** | **BAF** | **PBAF** | **ncBAF** |
| Conserved | Arp7 | | ACTB | | |
|  | Arp9 | | ACTA2 | | |
|  | Rsc6 | Swp73 | SMARCD1/2/3 | | |
|  | Rsc8 | Swi3 | SMARCC1/2 | | |
|  | Sfh1 | Snf5 | SMARCB1 | | |
|  | Sth1 | Snf2 | SMARCA2/4 | | |
|  | Rsc9 | Swi1 | ARID1A/B | ARID2 | GLTSCR1 |
| Metazoan Specific |  |  | SMARCE1, BCL7, SS18 | | |
| Yeast Specific | Npl6 | Swp82 |  | | |
|  | Rtt102 | |  |  |  |
| Complex Specific | Htl1, Ldb7, Rsc1/2, Rsc3, Rsc30, Rsc4, Rsc58 | Snf6, Snf11, Taf14 | DPF2 | PHF10, BRD7, PBRM1 | BRD9 |
